# HIV and Solid Organ Transplantation: Where Are we Now

**DOI:** 10.1007/s11904-019-00460-7

**Published:** 2019-09-04

**Authors:** Jean Botha, June Fabian, Harriet Etheredge, Francesca Conradie, Caroline T. Tiemessen

**Affiliations:** 1 Wits Donald Gordon Medical Centre, Department of Internal Medicine, School of Clinical Medicine, Faculty of Health Sciences, University of the Witwatersrand, 7 York Rd, Parktown, Johannesburg, 2193 South Africa; 2 Clinical HIV Research Unit, School of Clinical Medicine, Faculty of Health Sciences, University of the Witwatersrand, 7 York Rd, Parktown, Johannesburg, 2193 South Africa; 3 Centre for HIV & STIs, National Institute for Communicable Diseases, 1 Modderfontein Road, Sandringham, 2131, Private Bag X4, Sandringham, Johannesburg, 2131 South Africa; 4 School of Pathology, Faculty of Health Sciences, University of the Witwatersrand, 7 York Rd, Parktown, Johannesburg, 2193 South Africa

**Keywords:** Solid organ transplantation, HIV, South Africa, Living donor, HIV-positive-to-HIV-negative, Antiretroviral therapy

## Abstract

**Purpose of Review:**

We review the international evolution of HIV and solid organ transplantation over 30 years. We emphasise recent developments in solid organ transplantation from HIV-infected to HIV-uninfected individuals, and their implications.

**Recent Findings:**

In 2017, Johannesburg, South Africa, a life-saving partial liver transplant from an HIV-infected mother to her HIV-uninfected child was performed. This procedure laid the foundation not only for consideration of HIV-infected individuals as living donors, but also for the possibility that HIV-uninfected individuals could receive organs from HIV-infected donors.

**Summary:**

Recent advances in this field are inclusion of HIV-infected individuals as living organ donors and the possibility of offering HIV-uninfected individuals organs from HIV-infected donors who are well-controlled on combination antiretroviral therapy (cART). The large number of HIV-infected individuals on cART is an unutilised source of otherwise eligible living organ donors. HIV-positive-to-HIV-negative organ transplantation has become a reality, providing possible new therapeutic options to address extreme organ shortages.

## Introduction

Solid organ transplantation is the best therapeutic option for those with end-stage organ failure, most commonly of the kidneys, liver or heart [1–3]. Worldwide, the pool of human donors (living or deceased) falls far short of the increasing demand for organs, resulting in the deaths of many patients waitlisted for transplant. Efforts to increase the pool of available donor organs include donation after cardiac death (DCD), increased use of extended criteria or marginal organs, living donor programmes for kidney and liver donation, and split liver transplantation [4–7]. Currently, xenotransplantation, whilst appealing, has failed to overcome barriers to implementation and is not a viable option [8]. As organ shortages persist, the option of utilising living or deceased donor organs from people with chronic viral infections such as HIV and hepatitis C virus (HCV) has become a therapeutic reality. These advances have been facilitated through the availability of improved treatment options, such as direct-acting antivirals (DAAs) for HCV, and triple-combination antiretroviral therapy (ART) for HIV [9–11]. Presently, most organs from HIV-positive deceased donors have been implanted into HIV-positive adult recipients. There is scant literature on transplantation in the paediatric HIV-positive population [12–14].

HIV is a complex condition, not only due to its pathogenesis and symptoms, but also because of the social milieu surrounding it. It is associated with systemic stigma, which persists even in countries like South Africa (SA) that have robust HIV management and prevention programmes, as well as a vocal and committed activist community. In other countries, HIV-related stigma seems more prolific, and HIV is still often negatively associated with homosexuality, intravenous drug-use and promiscuity. It is this stigmatised framework that complicates the field of HIV and solid organ transplantation, because all decisions need to be considered in terms of the much broader and potentially harmful social implications for those involved, not to mention the medical ramifications.

The field of solid organ transplantation and HIV is rapidly evolving. Where we are now is that in 2017, our team at Wits Donald Gordon Medical Centre (WDGMC), part of the University of the Witwatersrand medical teaching complex in Johannesburg, SA, performed the first living donor liver transplant from an ART-suppressed HIV-infected donor mother to her HIV-uninfected child [15••]. This transplant is notable for being an intentional, controlled transplant of an HIV-positive donor organ in order to save the life of the recipient, something that had not been previously attempted. What makes this transplant particularly unique, however, is that we assumed HIV transmission to our recipient was a *fait accompli*, but this might not have occurred. This was the first report of a known HIV-positive person intentionally accepted as a living donor for any organ, worldwide.

In this fast-moving field, the utility of HIV-positive living donors is now being pushed further, and an HIV-positive living person recently donated a kidney to an HIV-positive recipient in the United States of America (USA) (https://www.scientificamerican.com/article/worlds-first-hiv-to-hiv-kidney-transplant-with-living-donor-performed-successfully/). With each bold move, the transplant community opens up new options for expanding the donor pool and enabling transplantation.

A recent excellent review has focused on the history and progress made in HIV-positive-to-HIV-positive transplantation, where pioneering work has been done [16•]. This current review extends the field, to consider specifically HIV-serodiscordant organ transplantation from HIV-infected donors to HIV-uninfected recipients. In this paper, we attempt to answer the question of ‘where are we’, currently, in solid organ transplantation and HIV. We review the legislative process governing HIV and solid organ transplantation over time, and how it has evolved. We then explore the potential for HIV-positive donation and outcomes for recipients who have received HIV-positive donor organs. Finally, we consider some of the new possibilities in HIV and solid organ transplantation for the future, especially in terms of diagnostic challenges that we now face.

## The History of Solid Organ Transplantation in HIV-Infected Individuals

**Fig. 1:**
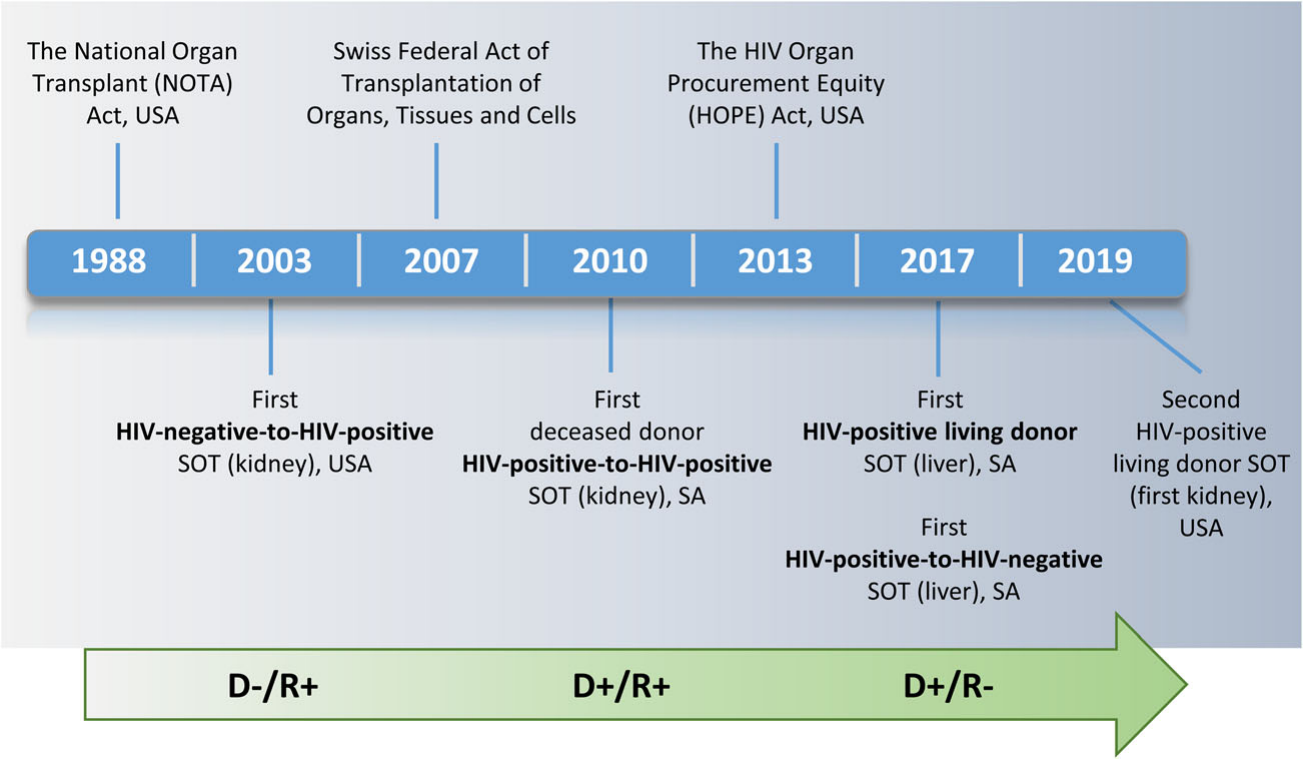
Timeline of key developments in the field of solid organ transplantation in the context of HIV infection. SOT, solid organ transplant; USA, United States of America; SA, South Africa; D, donor; R, recipient, −, HIV-negative; +, HIV-positive

Figure 1 depicts the key events that highlight progress in the field of solid organ transplantation and HIV. Prior to the emergence of the triple-combination ART in 1996, survival of HIV-positive patients receiving an organ transplant was inferior to that of their HIV-negative counterparts [17, 18], and the procedure was not widely performed. Since the advent of relatively widespread access to ART, HIV-infected patients are now accepted recipients of both HIV-infected and HIV-uninfected donor organs in specialised transplant centres worldwide. Studies suggest that HIV-infected recipients of HIV-uninfected solid organs have similar survival rates to those of HIV-uninfected recipients [19]. Transplant eligibility criteria for HIV-infected patients have also evolved, and HIV-positive status is no longer a contraindication for organ transplantation. HIV-infected transplant candidates are required to fulfil the same eligibility criteria as their HIV-uninfected counterparts, with some additional HIV-related stipulations [20].

Even with the advent of ART, however, the transplant community has viewed HIV in solid organ transplantation with scepticism. Some countries have adopted a more progressive approach through legislation than others. For instance, the Swiss Federal Act for Transplantation of Organs, Tissues and Cells has allowed transplantation of HIV-infected organs to HIV-positive recipients since 2007 [21].

The USA has a more complex history. In 1988, the use of organs from donors “infected with the etiologic agent for AIDS” was banned in the USA through the National Organ Transplant Act [19]. Recognising the growing need for transplantation in HIV-positive individuals, the HIV Organ Policy Equity (HOPE) Act was passed in the USA in 2013 and was largely a reaction to research taking place in South Africa [9, 22]. HOPE reversed the federal ban and mandated criteria for conducting research involving the transplantation of organs from HIV-positive donors, both living and deceased, to HIV-positive recipients. The intention was to increase the number of organs available to HIV-positive recipients. With this change, numerous new considerations need to be taken into account [9, 14, 19].

At Johns Hopkins University Medical Centre in the USA, the “HOPE in Action” clinical trial (https://clinicaltrials.gov/ct2/show/NCT03500315)—that evaluates HIV-positive-to-HIV-positive kidney and liver transplantation—is currently taking place. The results of this study will further explore the feasibility of utilising HIV-positive organ donors and how this could expand the donor pool. This would be to the benefit of both HIV-positive and HIV-negative individuals waiting for transplants.

## South Africa: Pioneering Through Necessity

It is noteworthy that the most comprehensive legislative measure in HIV transplantation, the HOPE Act of 2013 in the USA [23•], was primarily based on work done in Cape Town, SA, from 2008 onwards [24••]. Motivated by poor organ supply and lack of access to dialysis, Muller et al. [22] performed the first kidney transplants from HIV-infected donors to HIV-infected recipients in this setting. The use of organs from HIV-infected deceased donors was shown to be a safe, feasible alternative to dialysis [9].

South Africa has 7.2 million people living with HIV, 19% of the global burden, with 4.4 million receiving ART [25]. There seem to be two drivers of South Africa’s influence on international HIV-positive organ donor frameworks. The first is likely the unique nature of our HIV pandemic—which is of such scope as to warrant the consideration of HIV-infected people as donors in the face of extreme donor shortages. Moreover, the two-pronged National Strategy of PMTCT and ART for all infected with HIV in South Africa has called for specific and situational consideration of HIV-positive people as organ donors [26]. Secondly, South Africa’s transplant legislation—and accompanying health law—defers to the over-arching legal principle that informed consent is imperative for all such procedures. This applies to all medical and surgical interventions in this country regardless of their novelty [27]. In undertaking the first intentional transplantation of a liver segment from an HIV-positive living-donor mother to her HIV-negative child in 2017, our team had little local legal precedent from which to draw. The HOPE Act was relatively helpful in guiding donor selection; however, it does not address issues in selecting HIV-negative recipients. It is essential that national guidelines are formulated for HIV-positive donors and HIV-positive and HIV-negative solid organ recipients, and this process has commenced in South Africa.

## HIV-Associated Organ Disease

Broadly speaking, solid organ-specific manifestations of HIV can be divided into pathogenic effects from (i) HIV infection of the organ; (ii) opportunistic pathogens infecting the organ, particularly in the absence of ART and low CD4 counts; and (iii) consequences of ART. These effects, summarised in Table 1, include solid organs transplanted into HIV-positive recipients—most commonly liver, kidney and heart. They are far less frequent in combined organ transplants such as kidney-pancreas and liver-kidney [28]. Early initiation of ART with widespread access to treatment and long-term retention in care has largely mitigated the afore-mentioned effects of HIV and pursuant opportunistic infections on solid organs. Therefore, the “post-ART” era comprises a pool of potential living donors with well-controlled HIV whose survival is equivalent to those without HIV, despite the deleterious effects of some ART regimens. This is particularly relevant for countries with high HIV infection rates, good treatment access programmes and severe organ shortages, like SA.

**Table 1:** Common solid organ-specific manifestations of HIV before and after ART

Effect of HIV infection	Effect of opportunistic infections/other	Effect of ART	Potential organ donation
Heart and vascular system			
Vascular: • Premature atherosclerotic cardiovascular disease • Pulmonary arterial hypertension • Venous and arterial thrombosis • Arterial stiffness Non-vascular: • Myocarditis • Cardiomyopathy • Valvular disease (more with intravenous drug users)	Vascular: • Premature atherosclerotic cardiovascular disease (CMV, HSV1) • Arterial aneurysms (CMV, TB) Non-vascular: • Myocarditis (toxoplasmosis, CMV, cryptococcus, TB, HSV) • Intracardiac tumours (mostly Kaposi’s sarcoma and lymphoma) • Pericarditis (TB, Kaposi’s sarcoma)	Vascular: • Accelerated atherosclerosis^1, 2^ (lipodystrophy, dyslipidaemia, • Mitochondrial damage, insulin resistance) Non-vascular: • Ischaemic cardiomyopathy	Only deceased
Kidney			
Acute kidney injury HIV-associated kidney disease • Glomerular (i) HIV-associated nephropathy (ii) HIV-associated immune complex disease • Tubulointerstitial • Vascular Chronic kidney disease	• HBV • HCV • TB • CMV • Malignancy (Kaposi’s sarcoma, lymphoma) • Immune-reconstitution inflammatory syndrome • Comorbid chronic kidney disease (hypertension, diabetes)	Vascular: • Accelerated atherosclerosis^1, 2^ Nephrotoxicity: • Protease inhibitors^3^ (crystalluria, nephrolithiasis, interstitial nephritis, obstructive uropathy) • Tenofovir	Living and deceased
Liver			
AIDS-cholangiopathy Acalculous cholecystitis Vanishing bile duct syndrome HIV-associated liver injury/fibrosis	• Hepatotropic viruses: HBV/HCV/Delta virus /Hepatitis E • TB • CMV • Cryptococcus, histoplasma, extrapulmonary pneumocystitis • Primary hepatic malignancy - Hepatocellular carcinoma - Kaposi’s sarcoma - Non-Hodgkin’s Lymphoma • Secondary malignancy • Fatty liver disease (alcoholic and non-alcoholic) • Nodular regenerative hyperplasia • Immune-reconstitution inflammatory syndrome	Drug-induced liver injury (ART and non-ART related)	Living and deceased

^1^Protease inhibitor regimens: mostly ritonavir, indinavir, and amprenavir cause upregulation of CD36-dependent cholesteryl ester. ^2^Non-nucleoside reverse transcriptase inhibitors: through mitochondrial toxicity. ^3^Mostly seen with indinavir and atazanavir

### HIV-Infected Individuals as Living Donors

In living donation, wellbeing of the donor is paramount and either a single kidney or a segment of the liver may be utilised. Good evidence from healthy HIV-negative donors confirms that one can live a normal life with one kidney and the liver’s capacity to regenerate ensures adequate liver function after donation.

Specifically relating to the kidney, theoretical concerns have been raised that even with well-controlled HIV on ART, HIV-associated kidney disease (Table 1) may compromise the donor’s remaining kidney function. However, there are no data to confirm or refute this. It could be argued that if there was no evidence of HIV-associated kidney disease prior to transplant and donors remain on ART after donation, future HIV-related compromise of kidney function is unlikely. Recently, the first HIV-positive living donor kidney transplant was successfully performed in the USA. Longitudinal follow-up will begin to provide some answers.

In relation to liver donation, living donor liver transplants from HIV-positive donors in the USA have not been performed due to concerns of a possible increased risk for surgical complications. However, this is contrary to a substantial body of evidence confirming equivalent surgical outcomes in HIV-negative and well-controlled, HIV-positive individuals [28]. Inferior outcomes of HIV-positive recipients after liver transplant in the subgroup with HIV and HCV co-infection have also been flagged; however, these outcomes were prior to the advent of DAA’s for treatment of HCV [28, 29]. Other concerns pertain to potential risks of hepatic injury from HCV/HCV co-infection and/or ART-related hepatotoxicity that might compromise a living donor after transplant, placing the donor at higher risk for end-stage liver disease [19, 30].

## Transplantation Outcomes in HIV-Positive Recipients

US and European studies have demonstrated favourable outcomes in HIV-infected recipients of uninfected kidneys and livers. The relative risk of rejection and graft failure is higher than in HIV-negative patients, but the HIV itself does not seem to increase overall mortality [10, 31].

Stock et al. [32] reported that 150 HIV-positive patients who received organs from HIV-negative donors demonstrated good survival and that HIV-positive patients could safely undergo organ transplantation. Muller et al. [9] described reasonable outcomes in the transplantation of 27 HIV-infected recipients with HIV-infected deceased-donor kidneys. Patient survival at 1 and 3 years were both 84% while 5-year survival was 74%. Graft survival at 1, 3 and 5 years was 93%, 84% and 84%, respectively. These findings were similar to those in HIV-infected recipients receiving HIV non-infected organs. Whilst rejection rates were slightly higher in this cohort compared to Stock et al. [32], patient and graft survival was comparable at 1 and 5 years, respectively.

A large, multicentre Italian study described long-term transplant outcomes of liver, kidney, heart, lung and combined kidney-pancreas transplants in HIV-infected recipients [14]. Twenty-nine transplant centres participated, and 257 qualifying solid organ transplants were performed during the study period. Kidney and liver transplants were most common. The inclusion criteria for transplantation in HIV-positive recipients were the same as those in HIV-negative recipients, with additional criteria being CD4 count greater than 200 cells/mm^3^, viral suppression on ART and the absence of AIDS-defining events. The primary cause of death post-transplant was HCV co-infection, particularly in liver and kidney recipients. HCV/HIV coinfection outcomes were reported to be poorer than HIV mono-infected patients. HCV infection was also more aggressive in the HIV/HCV coinfected group than it was in the non-HIV infected group. Specifically, survival in liver transplant patients with HCV co-infection was only 50% while kidney transplant patients showed better outcomes with 95% survival. The poorer outcome in liver patients appeared to be associated with chronic HCV, and to a lesser extent HBV infection. With the advent of DAA’s, these figures are expected to improve in the future.

Data from the USA appears to demonstrate better survival than data from Italy [33]. One multicentre study compared data on HIV-infected transplant recipients to age- and race-matched data in HIV-uninfected recipients obtained from the United Network for Organ Sharing (UNOS) [34]. There was no statistical difference in the cumulative survival rate at 1, 2 and 3 years in HIV-infected patients (87%, 73% and 73%) as compared to the HIV-uninfected group (87%, 82%, 78%). In the HIV-infected group, poorer outcome was associated with HCV co-infection, CD4 counts of less than 200 cell/mm^3^ prior to transplantation and poor tolerability of ART post-transplantation.

## HIV-Positive-to HIV-Negative Organ Transplantation

### Unintentional Organ-Associated HIV Transmission

There are few reported examples of inadvertent HIV transmission events, where deceased organ donors were HIV-infected, but this was not detected on screening tests pre-transplant [35–40]. One article cited laboratory error as the cause of unintentional transplants from an HIV-positive donor to five HIV-negative recipients [41]. In this case, the heart, liver, both kidneys and a single lung were implanted. All recipients received ART within 48 h post-transplant. At 4 years post-transplant, all recipients were still on ART and demonstrated undetectable HIV viral load and CD4 > 200 cells/mm^3^. No opportunistic infections were reported. Graft and patient survival was 100%.

Since the inception of our paediatric liver transplant programme at WDGMC in 2005, we have been involved in one case of inadvertent HIV transmission. We observed seroconversion in a paediatric patient who received a liver graft from a deceased donor in the window period for HIV infection at time of death (unpublished). The recipient of the HIV-infected donor liver at our centre is doing well and stable on ART.

### Intentional HIV-Positive-to-HIV-Negative Transplantation

HIV-positive-to-HIV-negative transplantation currently presents an ethical dilemma, because the primary objective should be procuring a disease-free organ for transplantation. The notion of implanting an infected organ, especially in the context of HIV, is met with shock and disbelief. However, the context is vital. Worldwide, but especially in developing countries like SA, there is a very limited pool of donor organs available for patients with end-stage organ failure. Children are particularly vulnerable in this regard because the need to size-match organs requires paediatric donors, which are few and far-between. Furthermore, with the success of the PMTCT programme, HIV-negative children are being born to HIV-positive mothers. Should these children require liver transplantation, their best chance of success is a related living donor. Many children succumb to organ failure before they have been transplanted. At WDGMC, 15–20% of children listed for liver transplantation die on the waiting list. For example, 72 children were listed for liver transplantation at the beginning of 2018. By the end of 2018, 22 remained on the list. During 2018, we transplanted 42 children, 12 children died and the remainder were removed from the waiting list because they recovered, became un-transplantable or presented with nutritional challenges that needed to be managed before re-listing. This scenario grounded the decision taken by our team to undertake the world-first living donor liver transplant from an HIV-positive mother to her HIV-negative child. We faced numerous ethical issues, which we carefully considered. Ultimately, it was concluded that it was in the best interests of the child to live with HIV, rather than to face certain death resulting from complications of end-stage liver failure due to biliary atresia [42•].

The transplant was undertaken as part of a research study, and Institutional Review Board (IRB) approval from the Wits Human Research Ethics Committee (Medical) [Clearance number M170290] was obtained prior to the procedure. Engagement with the IRB, and experts across numerous relevant disciplines, allowed us to consider the ethical implications from many different angles. A separate article detailing the ethics of the procedure has been published [42•]. The primary ethical issues we faced involved protecting the autonomy of the mother as donor, as she had on several occasions expressed a wish to donate despite her HIV status and with full knowledge of the transmission risk to her child. In this context, it was vital that the mother was carefully informed and counselled about the additional risks and the many unknown variables we faced. To this end, our Independent Donor Advocate (IDA) was invaluable in promoting the mother’s autonomy and communicating with the transplant team on her behalf.

Weighted against the mother’s autonomy were the best interests of her child, too young to give consent to the procedure, but ultimately the one who would live with the physical and psychological consequences. At the time of the transplant, it was unanimously agreed that saving the child’s life, whilst potentially transmitting HIV simultaneously, was in the child’s best interests. However, we did not expect the ambiguous nature of our HIV test results, which have raised new ethical issues such as the merits of an Antiretroviral Treatment Interruption and factors surrounding disclosure. In the face of these uncertainties, and as we navigate this landscape, we continue to consult widely in order to best manage these ethics quandaries, bearing in mind that the best interests of the child are paramount in our decision-making process.

Whilst not a donor, we invited the father—as well as the mother—to ultimately consent to the procedure, as both parents are responsible for caring for the child into the future. At this stage, that future is uncertain with regard to HIV status and determining the nature of ongoing diagnostic investigations and management.

Because HIV infection can be effectively controlled with ART, related virally suppressed living donors could potentially donate their organs as readily as HIV-uninfected individuals do. If HIV infection emanating from the transplanted organ can be controlled by ongoing provision of ART with good adherence, recipients may remain virally suppressed and thus have a similar quality of life to HIV-uninfected patients. Importantly, it is now well established that HIV infection is not accelerated by immunosuppressive therapy post-transplantation, if ART is appropriately initiated [43]. HCV-positive-to-HCV-negative organ transplants in the context of the provision of DAAs are now conducted in many US centres, highlighting how quickly transplantation options evolve. In our case, the mother was virally suppressed on ART for at least 6 months prior to donation with a CD4 count > 200 cells/mm^3^, and the recipient was started on ART the evening before transplantation to reduce the risk of acquisition of HIV infection. Both remain on ART.

Several landmark studies suggest that successful ART translates to zero risk of sexual transmission, supporting the U = U (undetectable = untransmissable) dictum [44, 45, 46••]. A donated organ as the source of HIV exposure, albeit from a virally suppressed patient, presents a very different scenario and we cannot assume U=U in this context. To inform how one might reduce the risk of transfer of HIV with solid organ transplantation, more studies are required to understand HIV persistence and compartmentalization and the immune environment in different organs of ART-suppressed individuals. Whether a longer course of preventative ART given to the recipient might further reduce the risk of infection is also not known. Furthermore, it is not known how the very different environment once grafted in the HIV-negative recipient host will impact on these same features. Given the diagnostic challenges in this setting (described below), answering these questions requires knowledge of the HIV infection status which can only be unequivocally determined off ART.

## Diagnostic Challenges and Future Research Needs: Controlled HIV-Positive-to-HIV-Negative Organ Transplants

Standard diagnostic tests for determining HIV infection measure HIV-specific antibodies, or HIV antigens, plasma HIV RNA or HIV cell-associated DNA. The WDGMC case was seropositive at 49 days post-transplant, which was the first time point tested after surgery [15••]. Although these responses have waned subsequently, HIV-specific antibodies remain detectable to date. However, we propose this response likely represents a maternal memory response to HIV rather than a de novo response produced by the child in response to HIV. Mechanistically, it is possible that recipient responses could be sensitised by passive transfer of HIV antibodies bound to Fcγ receptors on the mother’s immune cells or actively produced by mature maternal liver resident B cells or B cells transferred with the graft. As antibodies can engage multiple arms of the immune system through their Fc receptors and antigen presentation functions [47], any induction of recipient responses would be expected to be dependent on sufficient presence of HIV antigens and sufficient levels of maternal antibodies. It can be argued in our WDGMC child case that both are insufficient in quantity to appear to be a major factor in this regard.

Several studies support the transfer of donor-specific response to recipients. Cases of donor-to-recipient transfer of peanut allergy support donor-specific transfer of allergic responses [48–50]. A study of HCV-negative patients who received kidney allografts from donors who were HCV-antibody positive but nucleic acid negative (Ab+/NAT) found 14 of the 32 patients (44%) seroconverted following transplantation but were HCV RNA negative [51], suggesting the transfer of organism-specific immune responses that are not necessarily associated with acquisition of infection in the recipient. Another recent study highlights the early emergence and donor origin of anti-HCV antibody responses in recipients of HCV-infected organs [52]. Further complexities in interpreting the recipient serological responses have been highlighted by Nel et al. [53] and include possible decreased and delayed response rates and seroreversion as evidenced in response to vaccines and other infections [54–58]. The latter effects are all likely consequences of immunosuppressive treatment.

The development of detectable HIV-specific antibodies upon HIV encounter, ordinarily driven by a sustained period of viremia following acquisition of HIV infection, is compromised by early administration of ART. In adult studies of very early ART (Fiebig 1), the associated lack of detection of virus and of HIV-specific antibodies did not preclude rapid viral rebound when ART was stopped in 8 Thai patients [59], or rebound despite remission of 7.4 months in a single case with a very low peak HIV RNA of 22 copies/ml [60]. A substantial proportion of early treated HIV-infected children are found seronegative when tested years later [61–63].

These collective findings highlight that one cannot convincingly determine the HIV status of an uninfected recipient receiving an organ allograft from an HIV virally suppressed positive donor. With very early ART, it is unlikely that the recipient would develop a detectable HIV antibody response. Likewise, lack of detection of HIV RNA or cell-associated HIV DNA with highly sensitive tests cannot guarantee lack of HIV-1 infection as small numbers of latently infected cells below the level of detection of assays may be present and fuel viral rebound if ART is stopped. How immunosuppressive drugs might impact on viral rebound if the recipient is in fact infected is also unknown and potentially a concern. With ongoing advances in the field of immunomodulation and solid organ transplantation in attempts to overcome complications of immunosuppression and to enhance graft survival [64], such innovations may also be of benefit in the HIV solid organ transplant arena.

The setting of the WDGMC case, and future cases the team plans to undertake as part of an IRB-approved research project (Clearance number M171035] [15••], provides us with an informative and unusual (parenteral) mother-to-child transmission model that can be utilised to address very early events in the HIV exposure-infection continuum in children. This experience has raised many questions concerning the likelihood of acquisition of HIV in the recipient when the organ donor is HIV-infected and virally suppressed, and ART is given to the recipient prior to transplantation. As things currently stand, the only way to definitively establish HIV infection in the recipients of HIV-positive-to-HIV-negative transplantations would be to stop antiretroviral therapy with close monitoring of possible recrudescent infection. However, much remains to be understood concerning this unique setting of HIV exposure/infection and infection risk. Risk and benefit of any such an intervention would need to be weighed up very carefully and on a case by case basis.

## Conclusions

In this review, we have highlighted recent advances in the field of solid organ transplantation and HIV. One of the most notable findings is that controlled solid organ transplant from HIV-positive donors to HIV-negative recipients has been interrogated to a very limited extent. The HOPE Act does not address this type of transplantation and, if amended, may open-up additional therapeutic options for transplant in the USA. Clearly, HIV is still highly stigmatised internationally, and this may prevent lawmakers from considering serodiscordant transplants as an option. Once again, South Africa has taken the lead—out of necessity but possibly also because we have been desensitised to HIV to a greater extent than the rest of the world. We make every effort to view HIV as a chronic, manageable disease, and we aim to assist HIV-positive people in accessing healthcare services regardless of their status. It is hoped that this poses a challenge to established preconceptions of HIV which still linger, and which may prevent policy-makers from fully exploring such options. In the context of extreme organ scarcity, this position is not without its merits, because it allows us to save the lives of children who would otherwise die awaiting an organ transplant. As this field expands globally, increasing utilization of virally suppressed HIV-infected donors could expand the options for patients who are in desperate need of organs.
